# Tobacco and the Escalating Global Cancer Burden

**DOI:** 10.1155/2011/408104

**Published:** 2011-08-18

**Authors:** Richard F. Oppeltz, Ismail Jatoi

**Affiliations:** Department of Surgery, The University of Texas Health Science Center, Mail Code 7738, 7703 Floyd Curl Drive, San Antonio, TX 78229, USA

## Abstract

The global burden of cancer is escalating as a result of dramatic increases in the use of tobacco in the developing world. The use of tobacco is linked to the development of a broad variety of cancers, mainly lung cancer, the single most common cancer in the world. Tobacco smoking-attributable deaths extends beyond cancer and include stroke, heart attack and COPD. Widening disparities in cancer-related mortality have shifted towards a more dramatic burden in the developing world. Appropriate interventions must be implemented to reduce tobacco use and prevent global mortality that has escalated to epidemic levels. Tobacco control policies, including public health advertisement campaigns, warning labels, adoption of smoke-free laws, comprehensive bans and tax policies are highly effective measures to control tobacco use. Clinicians and academic institutions have to be actively committed to support tobacco control initiatives. The reduction in cancer related morbidity and mortality should be viewed as a global crisis and definitive results will depend on a multilevel effort to effectively reduce the burden of cancer, particularly in underprivileged regions of the world.

## 1. Introduction

The global burden of cancer is escalating, largely due to dramatic increases in the use of tobacco in less developed nations [1]. Indeed, overall cancer rates appear to be increasing in developing countries, even while they remain generally stable or show small decreases in many industrialized countries [2, 3]. Thus, global changes in tobacco use may eventually produce large disparities in cancer-related mortality rates between the developed and less developed countries of the world [4].

The purpose of this paper is to highlight the trend towards increased tobacco use and the increasing cancer burden in developing countries and suggest steps that might be taken to reverse this alarming trend. 

Tobacco was widely used by the Mayans and other Native Americans well before Christopher Columbus introduced it to Europe in 1492. Within 150 years after its introduction to Europe, tobacco use was common throughout the world. Over the centuries, the methods of tobacco usage have changed considerably. In the 18th century, snuff held sway; the 19th century was the age of the cigar; the 20th century saw the rise of the manufactured cigarette and with it a greatly increased number of smokers [5]. Although the worldwide use of tobacco has steadily increased since the 16th century, early public statements showed its disapproval as stated by James I of England in his Counterblaste to Tobacco in 1604: “Smoking is a custom loathsome to the eye, hateful to the nose, harmful to the brain, dangerous to the lungs, and in the black, stinking fume thereof nearest resembling the horrible Stygian smoke of the pit that is bottomless.” Thus, even though its health risks have been acknowledged for centuries, tobacco use throughout the world continues to increase.

Most people who use tobacco regularly do so because of their addiction to nicotine, a major component of cigarettes. Although the majority of users express a desire to reduce their use or stop entirely, overcoming the addiction is difficult and may require both pharmacologic and behavioral treatments. Recent research has clarified the addictive nature of nicotine, and it appears to be similar to that of the opiates, cocaine, or other illicit drugs [6, 7].

Environmental factors likely also contribute to the increased use of cigarettes. For many, the behavior of smoking is not simply a matter of addiction, nor one of poor self-image, but also occasionally to underlying mental illness [8].

## 2. Tobacco-Associated Cancers

The association between tobacco and lung cancer was initially demonstrated by Doll and Hill in the 1950s in the UK [9]. Since then, additional case-control studies [10] and prospective cohort studies [11] have all affirmed the association between tobacco and the development of lung cancer. Indeed, lung cancer was rare in the early decades of the 20th century, but with the increase in smoking tobacco, it has become an alarming epidemic.

The tobacco hazard, although clearly linked to the development of lung cancer, also causes an increased risk of several other cancers, notably oral, larynx, pharynx, esophagus, stomach, liver, pancreas, kidney, bladder, uterine cervix cancers, and myeloid leukemia [12]. 

There is a clear dose-response relationship between cancer risk and tobacco use. A lifetime smoker has a risk 20–30 times greater than of a nonsmoker [13]. More than 4,000 chemicals have been identified in tobacco smoke, and some 60 are known or suspected carcinogens [14]. Each cigarette brings approximately 10 mg of soot, tar, ash, phenols, benzpyrene, hydrogen cyanide, formaldehyde, and radioactive polonium 210 into the lungs of the smokers [5].

## 3. The Global Problem

Worldwide, cancer is responsible for 1 out of every 8 deaths (more than HIV/AIDS, tuberculosis and malaria combined [15]), and tobacco use is responsible for one-third of all cancer-related deaths [16]. The International Agency for Research on Cancer (IARC) estimates that there were approximately 12.7 million new cases of cancer diagnosed in the world in 2008, and 7.6 million deaths attributed to it [17]. 

Furthermore, tobacco is responsible for 87% of all deaths attributable to lung cancer [18], now the single most common cancer in the world. It is estimated that by 2030 lung cancer will be the sixth most common cause of death in the world, compared with its current ranking of ninth [19]. 

Tobacco smoking-attributable illness extends beyond cancer and includes stroke, heart attack, and COPD. Indeed, total tobacco-attributable deaths are projected to rise from 5.4 million in 2005 to 6.4 million in 2015 and to 8.3 million in 2030 [19], with an estimated 600,000 deaths attributable to second-hand smoke [20]. These projections are based on models that show a three- to four-decade lag between the rise in smoking prevalence and the increase in smoking-attributable mortality that results from it [21]. 

Yet, if appropriate measures to control tobacco were implemented, a large proportion of these deaths could be averted. A number of indirect methods to estimate the mortality attributable to tobacco use have been developed; however, limitations related to specific countries and age population groups have been noted in the literature [22–25]. Unless there is widespread cessation of smoking, approximately 450 million deaths will occur as a result of smoking by 2050, and most of these will occur in current smokers [26]. 

For instance, the global burden of lung cancer has shifted significantly from approximately 31% of cases occurring in developing countries, to now up to 55% occurring in these countries [27]. This makes the widening disparities in cancer-related mortality between developed and developing countries even more tragic. Indeed, the World Health Organization (WHO) estimates that 40% of all cancers diagnosed today could have been prevented, partly by maintaining healthy diet, promoting physical activity, and preventing infections that may cause cancer, but largely through tobacco control [28].

Although the contribution of tobacco use to disease and death is well known, less attention has been given to the ways in which tobacco increases poverty and broadens social inequalities [29]. For example, in Vietnam, the amount spent on cigarettes ($US 416.7 million) is enough to feed 10.6–11.9 million people per year [30]. Furthermore, it has been reported that in China, poor individuals may spend up to 60% of their income on cigarettes, taking away money from food and children education [31]. Serious environmental problems are also associated with tobacco production, which requires the greater use of fertilizers and pesticides and massive deforestation for curing tobacco leaves. One tree is wasted for every three hundred cigarettes produced, and it is estimated that land used for tobacco cultivation worldwide could potentially be used to feed about 10 to 12 million people [32]. As in other agricultural sectors, child labor is prevalent in the tobacco farms, particularly in the poorer areas, where up to 80% of children are missing school and/or undertaking hazardous tasks due to farm work [33].

Currently, smoking imposes a huge economic burden in developed countries, responsible for 15% of the total healthcare costs [34]. Developing countries, with higher population growth rates, are not prepared to cope with such increases in their healthcare expenditures. 

## 4. Tobacco Industry

In many industrialized countries, tobacco use appears to be declining, largely due to the diligent efforts of public health officials. In response to these declines, the tobacco industry is now targeting third world markets, not only to expand their markets, but also as a source of less expensive tobacco.

The tobacco industry includes some of the most powerful transnational companies in the world. These companies sell about six trillion cigarettes each year, which accounts for the largest share of manufactured tobacco products, comprising 96% of the total value sales [35]. The industry is highly concentrated within a handful of firms. The global tobacco market, valued at US$ 378 billion, grew by 4.6% in 2007 and by the year 2012 is expected to increase another 23%, reaching US$ 464.4 billion [36]. China is the biggest tobacco market, based on total cigarettes consumed. There are some 350 million smokers in China who consume around 2,200 billion cigarettes a year, or about 41% of the global total. However, the industry in China is state owned. Outside of China, the four largest publicly-listed international tobacco companies account for about 46% of the global market. Although the tobacco companies have experienced declines in profits in industrialized countries, their overall profits are increasing, driven by world population growth, particularly in Asia. The tobacco companies have reacted to stagnating demand on their traditional markets in basically three ways: consolidation (dominating the business by few but very influential companies), diversification (by producing low- and high-quality cigarettes and geographic diversification), and increasing productivity [37]. Worldwide, even if the prevalence of tobacco use falls, the absolute number of smokers will increase due to the huge population of the developing world [38]. 

The giant multinational cigarette companies generally find that the political and social climate in the developing world is conducive to their business [39]. Governments in these countries use tobacco taxation as a source of much needed revenue and, therefore, do very little to discourage tobacco use. Furthermore, people in the developing world are generally much less knowledgeable about the health risks associated with cigarettes, and there exist very few antismoking campaigns, with tobacco products often carrying no health warnings [40]. In Pakistan, for example, health warnings, even if available, tend to be very vague and poorly understood [41]. High-tar cigarettes, banned in developed countries, continue to be sold in the developing world. For example, nicotine contents for Indonesian kreteks or clove cigarettes are between 1.7 and 2.5 mg per stick compared with <0.05 and 1.4 mg per stick for cigarettes sold in the USA [42].

Yet, the tobacco companies are continuing their marketing efforts in the industrialized countries as well. Although no other consumer product is more dangerous or kills as many people as does tobacco, it still remains the most advertised product in the USA, with estimated advertising expenditures in the tens of billions of US dollars every year [35].

Facing global antitobacco forces, the tobacco industry is already moving beyond what they refer to as “light” and “mild” cigarettes to a new generation of tobacco products referred to as “potential-reduced exposure products” (PREPs) [43]. These products, which have been in development for decades, are the next step after filters and low-delivery “light” and “mild” cigarettes. The essential idea behind PREPs is that they will deliver the levels of nicotine required for a smoker's addiction with less (but some) of the toxins associated with smoking [44]. Yet, these products clearly are associated with alarming health risks, downplayed by the tobacco industry.

In May, 1999, researchers of the World Bank's Health, Nutrition and Population sector published a paper entitled “Curbing the Epidemic: Governments and the Economics of Tobacco Control.” This document concluded that tobacco control is not only good for health, but also good for the economy. Yet, multinational tobacco companies have attempted to use their own “economic impact studies” to convince governments that, contrary to the World Bank's conclusion, tobacco use benefits the economy. Thus, the tobacco industry continues its diligent efforts to undermine any threat to its profits. There are several investigators who have argued that the tobacco industry propagates disinformation, manipulates research, and generates faulty information concerning the effects of tobacco use and second-hand smoke [7, 45].

## 5. The Growing Problem in the Developing World

Worldwide, cigarette consumption is increasing at a rate of about 3% annually. In Asia, Southern and Eastern Europe, and developing countries, tobacco use is increasing at about 8% per year. Yet, in some industrialized countries, smoking rates are decreasing at about 1% a year, largely due to the implementation of significant anti-tobacco programs. As with all other epidemics involving a major behavioral component, the exact timing, duration, and magnitude of the smoking epidemic will vary significantly from one country to another. In China and many other developing countries, the rate of tobacco-related deaths is rising rapidly. China is now beginning to face the detrimental consequences of tobacco use, as many millions of individuals who began smoking in adolescence are now aging. Yet, it will be around 2030 before the epidemic of tobacco-related deaths peaks in China at the level achieved in the United States in 1990 [46]. Indeed, lung cancer rates in China have already been increasing about 4.5% a year. These trends reflect significant policy deficiencies towards tobacco use in developing countries.

**Figure 1:**
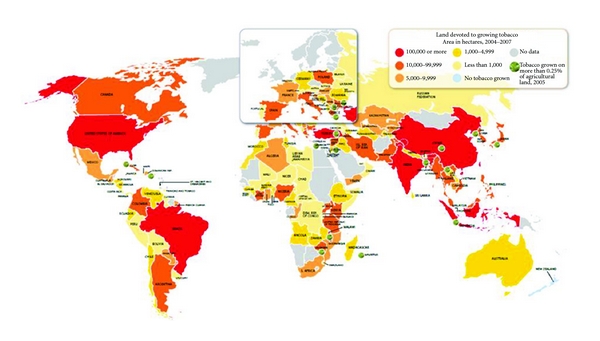
World land devoted to growing tobacco. The Tobacco Atlas, third edition. “Reprinted by the permission of the American Cancer Society, Inc., *The Tobacco Atlas, 3rd Edition.* American Cancer Society 2009, http://www.cancer.org/. All rights reserved.”

Most cigarettes are now consumed and produced in Asia. China alone produces close to 40 percent of world total, followed by India, Brazil, and USA (Figure 1). Neither tobacco nor cigarettes are a homogeneous product. Different conditions in the tobacco growing areas, (type of soil, rainfall, irrigation, and climate) handling and processing, ultimately will influence the quality of the leaf and the smoking product. Most manufacturers use a blend of different tobaccos in their product. However, the tobacco's leaf quality and additive contents will affect the particular taste of a cigarette brand and certainly the price [47]. 

The increasing incidence of cancer in developing countries reflects a transition in the global burden of disease away from one previously dominated by infectious diseases. This shift is also partly due to the ageing of the population and public health interventions such as vaccinations and the provision of clean water and sanitation in the developing world, all of which have served to reduce the burden of infectious diseases. 

Also some of the environmental, social, and structural changes linked to the transformation of a country from agrarian to industrial and then to a postindustrial state may lead to increased longevity in the population. As cancer is more common in the older age groups, cancer rates are expected to increase accordingly.

Yet, public health interventions can effectively lower the cancer rates. Low- and middle-income countries, faced with the tobacco epidemic, can learn from the tobacco-control successes in high-income countries by enacting cost-effective tobacco-control policies. Such policies can effectively reduce the burden of cancer.

## 6. Global Approaches to an Escalating Cancer Burden

Many interventions (public health advertising campaigns, warning labels on tobacco products, etc.) that were developed in the industrial world to curb tobacco use should be urgently implemented in the developing world. Interventions to reduce tobacco use may not only avert a large burden of unnecessary deaths, but also save governments huge health care costs. To prevent death or morbidity from cancer, interventions should target behaviors or risk factors that are responsible for tobacco use, and these interventions should be cost effective [48]. 

The Disease Control Priorities Project (DCPP), a joint effort of the Fogarty International Center of the US National Institutes of Health (NIH), World Health Organization (WHO), and The World Bank, was launched in 2001. This project aims to assist decision makers in developing countries find affordable, effective interventions to improve the health and welfare of their populations [49]. 

The spirit of international cooperation is exemplified in The Tobacco Control Country Profiles database, a data collection initiative led by the American Cancer Society, the World Health Organization (WHO), and the Centers for Disease Control and Prevention. It represents a worldwide information system to support global tobacco control efforts [50].

The World Health Organization (WHO) has led international strategies to eradicate tobacco use. The WHO Framework Convention on Tobacco Control (FCTC), the first global treaty in response to the tobacco epidemic, adopted in 2003, sets the foundation for price and nonprice population-based control interventions to reduce both demand for and supply of tobacco products and provides a comprehensive direction for tobacco control policy at all levels (Table 1). As of October 2010, 172 countries have ratified the treaty, representing 87.3% of the world's population. Up to a 21% reduction in smoking prevalence can potentially be achieved by implementing important interventions, such as increased taxes on tobacco products, enforcement of smoke-free workplaces, controls on packaging and labeling of tobacco products, and a ban on tobacco advertising, promotion, and sponsorship [51].

The Tobacco Control Program of the WHO was developed in response to the globalization of tobacco use. It is based on the principles of the FCTC, provides data-supported effective measures for tobacco control at all levels, and launches an annual global report summarizing the most current status of the application of those strategies. In 2008, WHO introduced a package of principles under the acronym of MPOWER intended to assist in the country-level implementation of effective measures to reduce the demand for tobacco.  Table 2 summarizes those key points. 

There are dozens of more national and international nongovernmental organizations which address tobacco control as part of their activities and numerous additional partner organizations that promote tobacco control among their initiatives [35]. 

Despite significant improvements worldwide in cancer diagnosis and treatment, much still remains to be done [52]. Cancer is a global challenge. Health-oriented resources should be allocated to collect accurate cancer data [53]. In developing countries, cancer registries are perceived as a luxury and rarely provided sufficient resources. International scientific societies have defined standards for cancer data collection, starting with a hospital-based registry which can be the first step towards the formation of a population-based cancer registry. The major aim of a cancer registry is to produce and interpret data to develop country-specific research protocols and cancer control plans [54].

In high-income countries, comprehensive bans on all advertising, promotion, and sponsorship protect people from industry marketing tactics and decrease tobacco consumption by approximately 7%. It has been suggested that these preventive measures might be twice as effective in low and middle-income countries potentially reducing global cancer mortality rates [55, 56]. The Family Smoking Prevention and Tobacco Control Act, a United States federal law that gives the Food and Drug Administration (FDA) the power to regulate the tobacco industry, was signed into law on June 22, 2009 by President Barack Obama. The Tobacco Control Act requires that cigarette packages and advertisements have larger and more visible graphic health warnings (including nine new textual warning statements and color graphics depicting the negative health consequences of smoking) and a prohibition on the manufacture of products that use the terms “light,” “low,” “mild”, and similar descriptors [57]. 

Tax policies that raise the price of tobacco products are the single most effective approach for reducing demand, since consumption is highly influenced by the extent to which smokers can afford to purchase cigarettes [58]. Price increases are especially effective against the initiation of smoking in youth and motivating addicted smokers to quit [59]. A 10% price increase may cause a 4% drop in tobacco consumption in high-income countries and an 8% drop in low- and middle-income countries, in addition to increasing tobacco tax revenue [60]. Additional price cap regulations (wherein a cap is placed on the pretax cigarette manufacturers' price) limits excess profits for the tobacco industry and increases government revenue [61]. 

The magnitude of the price increase is one of the most important predictors of an intention to quit/smoke compared with the average cigarette price. However, the availability of alternative (cheaper) cigarette sources may reduce but would not eliminate the impact of higher prices/taxes on the expected intention to stop smoking [62]. Illegally sold cigarettes evade taxes, and indeed, smugglers put cheap cigarettes into the hands of those most vulnerable, the developing countries, where those activities have been rising exponentially. Tobacco has now become the world's most widely smuggled legal substance. The World Health Organization estimates that as many as 25% of all cigarettes sold in the world are smuggled. For the international gangs that organize the traffic, it is even more profitable than drug smuggling [63].

Cessation programs have been shown to provide benefits to certain populations [64]. Cessation programs have a role at all levels of the health workforce, including primary care, health specialists, and smoking cessation specialists. 

Adoption of smoke-free laws, included in the article 8 guidelines of the FCTC, has been shown to reduce hospital admission for heart attacks and results in an overall decrease in acute coronary events [65]. Multiple successful examples of countries and cities around the world that have implemented smoke-free laws support the fact that with adequate planning and resources, tobacco-free enforcement protect health and profits the economy [66]. Latin America remains at the forefront of global progress with Colombia, Guatemala, Paraguay, Peru, and Honduras recently added to that growing list [67]. The European Union is proposing a full-scale ban on branded cigarettes, forcing tobacco companies across the continent to sell their products in generic, plain packaging. Worldwide, 25 countries already switched from text to graphic health warnings [68].

International organizations and governments have found certain constraints and barriers to succeed in the war against tobacco: lack of adequate technical and financial resources and capacities for tobacco control; weakness or lack of effective national legislation on tobacco control; lack of public and media awareness of the harmful effects of tobacco use; tactics of the tobacco industry in hindering effective implementation of already adopted legislation or interference in the development of such legislation; lack of or insufficient political will or intersectoral cooperation in tobacco control [69].

Tobacco control policies implemented in high-income countries may not necessarily have a similar effect in low- and middle-income countries, and public health officials should consider this possibility when planning appropriate interventions [70]. In summary, the definitive results in public health improvement will depend on how aggressive a particular government is on implementing the elements of the WHO's FCTC.

## 7. Clinician and Academic Institution-Based Initiatives

Many of the cancers that pose the greatest threat to developing countries are directly linked to tobacco use. In developed countries, most patients have access to a full range of healthcare resources, including smoking cessation programs, but this is not the case in the developing world. In the developing world, primary care physicians and health workers will need to be more involved in cancer control through health promotion programs that emphasize the hazards of tobacco use and prioritize tobacco cessation. Moreover, many low- and middle-income countries will likely see greater increases in quality-adjusted life years (QALYs) through implementation of smoking cessation interventions, tax policies, bans of promotion and advertisement, and adoption of smoke-free laws, mainly because tobacco-related cancers are preventable, and specialty cancer care is often limited in these countries [15].

The role of the health professionals is critical in tobacco control. At the local level, brief clinical interventions should be implemented based on patient's willingness to quit. Strategies should be implemented to advise patients to quit, to reinforce their decision to quit, and identify those who are at risk for relapse to smoking, providing such individuals with counseling, pharmacotherapy, or both [71]. 

Unfortunately, in many countries, the prevalence of health professional smokers is similar to that of the general population. To set an example, health professionals should be urged to stop smoking [72]. Thus, the eradication of tobacco should become a priority for not only governments, but also medical schools and physicians.

Multiple studies have shown that there are differences in patient approach, assistance [73], and educational role between smoking versus nonsmoking physicians [74]. Smoking physicians benefit from practical assistance in quitting themselves and providing support to their patients [75]. In 2008, the U.S. Department of Health and Human Services launched a Clinical Practice Guideline, which summarizes the most updated recommendations in clinical treatments for tobacco dependence based on systematic review of evidence-based research that should be implemented by every physician [64]. Along the effective strategies available, clinicians also must be committed to follow a code of professional ethics regarding tobacco. Those should include

1. physicians not smoking,
2. make tobacco cessation assistance a routine part of oncology care,
3. establish all medical facilities to be 100% smoke-free,
4. teaching physicians should lead their students to never become smokers and train them in the principles of smoking cessation,
5. reject any involvement of the tobacco industry in financing research, training programs, or treatment services for patients,

Several international societies have trained medical and surgical oncologists as part of their effort to address the burden of cancer in developing countries. Available educational resources for clinicians in developing countries include the European School of Oncology (ESO), the International Campaign for the Establishment and Development of Oncology Centres (ICEDOC), the Global Core Curriculum in Clinical Oncology developed by the European Society for Medical Oncology (ESMO), and the American Society for Clinical Oncology (ASCO), among several others [76, 77]. 

Unfortunately, some academic institutions have received funds from the tobacco industry to support biomedical research. Universities and researchers must understand the motivation underlying such offers of support. By legitimizing the tobacco industry, universities risk their integrity, values, and public trust [78]. Academic institutions should therefore reject offers of funding from the tobacco industry.

## 8. Conclusion

The reduction of cancer-related morbidity and mortality in developing countries should now become an urgent global priority. Developing countries already have enormous limitations in resources and are unable to cope with an escalating cancer burden. Additionally, an escalating cancer burden in developing countries is not in the best interests of the developed world and should be viewed as a global crisis. Urgent efforts are now needed to curb the widespread use of tobacco and thereby effectively reduce the burden of cancer, particularly in underprivileged regions of the world.

## Figures and Tables

**Table 1:** Key policy provisions of the WHO framework convention on tobacco control [8].

FCTC article no.	Policy
6	Price and tax measures to reduce demand.
8	Protection from exposure to tobacco smoke.
9	Regulation of the contents of tobacco products.
10	Regulation of tobacco product disclosures.
11	Controls on packaging and labeling of tobacco products.
12	Programs of education, communication, training, and public awareness.
13	Bans on tobacco advertising, promotion, and sponsorship.
14	Programs to promote and assist tobacco cessation, and prevent and treat tobacco dependence
15	Elimination of illicit trade in tobacco products.
16	Measures to prevent the sale and promotion of tobacco to young people.
17	Provision for support for alternative crops to tobacco.
20	Provision for an epidemiologic monitoring system.
22	Cooperation among the parties to promote the transfer of technical and scientific expertise on surveillance and evaluation.

**Table 2:** World Health Organization MPOWER key points [1].

**M**onitor tobacco use and prevention policies.
**P**rotect people from tobacco smoke.
**O**ffer help to quit tobacco use.
**W**arn about the dangers of tobacco.
**E**nforce bans on tobacco advertising, promotion, and sponsorship.
**R**aise taxes on tobacco.
